# Online learning readiness and satisfaction in English as a foreign language at higher education context: the moderating role of engagement

**DOI:** 10.3389/fpsyg.2025.1602117

**Published:** 2025-06-19

**Authors:** Ertan Altınsoy, Serkan Boyraz

**Affiliations:** ^1^Department of Foreign Languages, School of Foreign Languages, Aksaray University, Aksaray, Türkiye; ^2^Department of Educational Sciences, Faculty of Education, Aksaray University, Aksaray, Türkiye

**Keywords:** EFL context, engagement in online learning, online learning readiness, online learning satisfaction, university students

## Abstract

**Introduction:**

With the rapid advancement of technology and the unexpected outbreak of COVID-19, educational institutions worldwide were compelled to shift to e-learning, especially in the field of foreign language instruction such as English as a Foreign Language (EFL). While online learning environments offer advantages like flexibility, accessibility, and interactivity, challenges persist in sustaining student engagement and ensuring satisfaction. This study was prompted by low student attendance in online EFL classes at a public university in Türkiye. The primary aim was to explore the mediating role of Engagement in Online Learning (EOL) in the relationship between Online Learning Readiness (OLR) and Online Learning Satisfaction (OLS).

**Method:**

The study employed a quantitative research design, involving a sample of 945 associate and undergraduate students enrolled at a state university in Türkiye. Standardized instruments were used to measure OLR, EOL, and OLS. Data were analyzed using correlation and mediation analyses, along with tests for moderation by demographic variables such as age and gender.

**Results and discussion:**

Findings indicated a medium-level, positive, and statistically significant relationship between students’ OLR and their OLS. A strong positive correlation was also found between OLR and EOL. Furthermore, engagement (EOL) was shown to significantly mediate the relationship between readiness (OLR) and satisfaction (OLS), suggesting that students’ active participation plays a crucial role in achieving satisfaction in online learning environments. Age was not a moderating factor in the readiness-satisfaction link, whereas gender was found to have a significant moderating effect. Additionally, a moderate, positive, and significant relationship was observed between engagement and satisfaction. These results highlight the importance of fostering engagement to enhance students’ online learning experiences and outcomes in EFL contexts.

## 1 Introduction

The unpredictable rapid advancement of technology penetrates deeply into every aspect of human life, revolutionizing the way we think, process information, and act (Bucăţa and Tileagă, 2024). The traditional boundaries of education, which restrict students and teachers with various contextual factors such as time, space, and resources, etc. have been overcome through e-learning environments and AI-driven adaptive learning environments that facilitate personalized learning at anytime and anywhere. In other words, e-learning platforms enriched with massive digital resources and innovative pedagogical approaches break down the constraints of traditional education and offer numerous opportunities for enhancing learning and teaching experiences.

Apart from ground breaking developments in technology in its immense impact in education, the unexpected COVID-19 outbreak and the immediate closure of educational institutions subsequently has been a compelling force for swift adoption and expansion of e-learning (Karagöz and Rüzgar, 2021) since traditional classrooms were no more accessible and the teachers and students were no more reachable. Although COVID-19 has posed various constraints in human beings’ daily lives, it could be argued that it has offered significant opportunities for the digital transformation of educational institutions, accelerating the integration of e-learning tools and platforms.

In line with these issues afore mentioned, e-learning has been considered as a significant alternative to conventional teaching mode (Ahmad et al., 2023; Çebi, 2023; Gros and García-Peñalvo, 2016) as it allows for accessibility, flexibility and interactivity (Liu and Yu, 2023) with diverse multimedia resources that can be modified or adapted depending on the students’ needs and individual styles (El-Sabagh, 2021) breaking geographical barriers (Al-Fraihat et al., 2020). E-learning, which has been used synonymously with online learning (Moore et al., 2011), has been defined as a digital instruction delivered synchronously or asynchronously that is intended to train and educate learners systematically (Clark and Mayer, 2024; Sun et al., 2008). Synchronous mode of delivery refers to instructor-led online learning designed for real-time interchange of information using various software tools (Clark and Mayer, 2024; Turnbull et al., 2021). On the other hand, asynchronous mode enables the participation of students and instructors at different times, allows them to access materials at their own pace, and engage with one another at different intervals through learning management systems or recorded lectures (Zeng and Luo, 2023). Since both modes have their own strengths and weaknesses in relation to each other, the common practice is designing a hybrid method that incorporates both modes of delivery in order to benefit from the advantages of both synchronous and asynchronous e-learning (Clark and Mayer, 2024).

Considering the strengths compared to traditional teaching models of teaching and the opportunities provided, there has been a global trend among higher education institutions in conveying content to the learners through online learning platforms (Gros and García-Peñalvo, 2016). Today, as a result of digitalization in education many higher education institutions have been offering courses online and making efforts to improve technical and technological infrastructure in order to increase the number of online courses to leverage accesibility. In line with this trend, online learning has rapidly evolved into a burgeoning academic field of interest. Many of the studies centres around the challenges and advantages of using e-learning systems from the perspectives of the learners (e.g., Al Rawashdeh et al., 2021; Gherheș et al., 2021; Maatuk et al., 2022) or the predictors of learner satisfaction in e-learning environments (e.g., Chen and Tat Yao, 2016; Rajeh et al., 2021; Nikou and Maslov, 2023).

Accordingly, we have also been witnessing a rush, especially in the last decade, by many public and private higher educational institutions to offer online language classes through various platforms. As a result, the scholarly works centred around the quality of language classes’ content conveyed through synchronous or asynchronous modes of teaching (Khojasteh et al., 2023), learners’ perceptions of online foreign language learning and teaching (Murday et al., 2008), the contribution of online language classes to the students’ receptive and productive skills, interactions, and learning outcomes (Harsch et al., 2021; Lin et al., 2017). However, this rapid transformation in higher education, which has also been accelerated by the COVID-19 outbreak, has brought forth various issues. E-learning does not merely mean transmission of online knowledge (Nikou and Maslov, 2023), and effective use of e-learning systems and maximizing the benefits which result in academic success depend on active and persistent endeavors of e-learning satisfaction and engagement of the students (Jung and Lee, 2018; Martin and Bolliger, 2018).

Online learning satisfaction (OLS) is a significant variable in determining the effectiveness of online learning systems and platforms. It is a multidimensional construct that is dependent on various factors including interaction, computer self efficacy, course structure, perceived usefulness, self regulation, motivation for learning etc. (Eichelberger and Ngo, 2018; Gray and DiLoreto, 2016; Landrum et al., 2021; Wei and Chou, 2020) which determine individual’s perception of the quality of online learning experience.

Engagement has a pivotal role in learning outcomes as portrayed in the literature (Hu and Hui, 2012; Kahu and Nelson, 2018). Engagement was defined as the “the students’ psychological investment in and effort directed toward learning, understanding, or mastering the knowledge, skills, or crafts that academic work is intended to promote” (Newmann, 1992, p. 12 as cited in Finn and Zimmer, 2012) and is consisted of three dimensions namely, behavioral engagement, cognitive engagement and emotional engagement (Fredricks et al., 2004). Behavioral engagement refers to a learner’s level of involvement in academic and social activities. Cognitive engagement is related to the idea of investment, incorporating careful consideration and willingness to invest the necessary effort to comprehend intricate concepts and excel in challenging tasks. Emotional engagement is related to the affective reactions of the learners towards teachers, classmates, institutions, etc. which is often characterized by the level of belonging and involvement. In parallel with digitalization in education, engagement played a crucial role in e-learning environments since the learners might have limited incentives, triggering behavioral, cognitive, and emotional engagement which could be directly related to the quality and effectiveness of online learning systems. Many studies revealed that engagement in online learning (EOL) is a significant indicator of the quality of online learning experience (Luan et al., 2023; Rajabalee et al., 2019).

Recent studies unrolls the close interplay between satisfaction and engagement of the learners in online learning platforms. Bolliger and Halupa (2018) reported that a notable positive correlation existed between student engagement and student outcomes, particularly concerning their perception of learning and satisfaction. Similarly, El-Sayad et al. (2021) found evidence of a significant and direct relationship between OLS and behavioral and emotional engagement of higher education students in Egypt. The study of Poon et al. (2024) also confirmed the mutual relationship revealing that positive perception of online learning contributes to e-learning engagement, thereby influencing e-learning effectiveness and satisfaction.

OLS is associated with a number of variables as mentioned before, and relevant research investigating factors affecting satisfaction has revealed that online learning readiness (OLR) is another critical variable in fostering satisfaction and academic success (Kumalasari and Akmal, 2021; Rafiee and Abbasian-Naghneh, 2021; Wei and Chou, 2020). OLR, proposed by Warner et al. (1998), is a multifaceted term, and it is possible to come across various definitions in literature (Clark and Mayer, 2016; Kaur and Abas, 2004; Rafiee and Abbasian-Naghneh, 2021; Warner et al., 1998). Based on these definitions, it could be briefly defined as the individual’s ability that consists of various sub-skills, including technical know-how of using digital resources and cognitive capacity to effectively engage in online learning. As it is broad in scope and multi-faceted in nature, several instruments were developed to conceptualize online readiness which revealed that OLR is consisted of certain dimensions as computer self efficacy, online communication self efficacy, self direction and initiative, self directed learning, motivation for learning, learner control etc. (Dray et al., 2011; Demir, 2015; Hung et al., 2010) and as shown in the literature, OLR is a prerequisite for success and academic achievement (Wang et al., 2023; Wei and Chou, 2020) and significant for effective and succesful online learning experience (Mirke et al., 2019; Rohayani and Kurniabudi, 2015).

There are different variables investigating learners’ OLR in the literature. Tang et al. (2021) investigated the predictor role of gender and education level of students from three higher academic institutions in Hong Kong in e-learning readiness and reported that no significant differences were observed in relation to gender, while postgraduate students had a higher level of readiness compared to undergraduate and sub-degree students. In another study, Yunusa and Umar (2021) observed the moderating effects of age and gender on higher education students’ perceptions of OLR and satisfaction and revealed a significant moderating effect within the relationship based on gender and age. Adams et al. (2022) also revealed similar findings, reporting that age, gender, ethnicity and level of education were significant variables impacting participants’ OLR, and significant differences were observed in students’ OLR depending on each variable.

Within this perspective, this study grew out of the concern related to the significantly low attendance of students to online foreign language classes at a public university in Türkiye, while discussing possible reasons behind this. Although nearly four thousand students have been enrolled in online foreign language classes in the 2023–2024 academic year, spring term, it was observed that approximately, just 1 percent of the students have been following the classes synchronously. Furthermore, those who attended do not effectively engage either. In this sense, it was hypothesized that low attendance could originate from students’ low satisfaction with online classes as revealed in the literature (Rakhmanov and Ulasbekov, 2021; Zaharia et al., 2022), which could be related to certain variables. Despite the growing body of research investigating factors impacting OLS, we argue that there remains a research gap exploring the underlying mechanisms, including OLR and EOL, which could be considered as key behavioral and psychological constructs for effective learning outcomes in online settings, where student engagement and attendance is low. Besides, existing studies have predominantly examined the relationships among OLS, OLR, and EOL in isolation, primarily focusing on whether direct or indirect associations exist between these variables. However, no studies have examined the mediating role of EOL in this relationship in the Turkish EFL context to the best of our knowledge. This gap is assumed to be critical when the influence of cultural, technological, and institutional factors of the Turkish educational context on online learning settings is considered. Moreover, the literature presents inconsistent findings concerning the influence of demographic variables such as age and gender as potential moderators in this framework, which suggests that further investigation is necessary to better understand how such variables may shape engagement and readiness in online learning environments. Addressing these gaps may explain the low attendance and engagement rates in online foreign language classes and contribute to existing literature.

Within this perspective, this study aims to examine the underlying mechanism by which OLR affects OLS. In this sense, it is proposed that (1) OLR is positively related to OLS, (2) OLR is positively related to EOL, (3) gender and age variables moderate the relationship between OLR and EOL, (4) EOL is positively related to OLS; and (5) EOL mediates the relationship between OLR and OLS.

## 2 Method

### 2.1 Participants and procedures

The participants of the study are associate and undergraduate students studying at a state university in the Central Anatolia region of Turkey. Students from all 81 provinces of Turkey are enrolled in this university, so there is diversity in terms of the demographic characteristics of the students. The necessary permission was obtained from the Ethics Committee of the university before the data collection process began, and the 1964 Declaration of Helsinki and its subsequent updates were followed throughout the entire process. Snowball sampling was used as the sampling method. The sample consisted of 445 students whom the researchers were teaching and other students who could be reached by the researchers. Snowball sampling was selected due to its practicality in accessing a wider student population beyond the initial teaching cohort. This method allowed the researchers to reach participants through peer networks, leveraging trust and social connections to encourage participation. To reduce sampling bias, the initial sample of 445 students was intentionally diverse, and recruitment chains were monitored to promote variety in the participants. Additionally, a large number of data collection tools (1.000) were distributed to support sample heterogeneity and mitigate overrepresentation of any particular group. Of these 1.000 data collection tools, 945 were returned and constituted the data set of the study.

It took about 10 min for the participants to complete the measurement tool, which consisted of three scales, namely readiness to online learning, engagement in online learning, and online course satisfaction. Of the 945 participants, 619 (65.5%) were female and 326 (34.5%) were male. Of the participants, 53 (5.6%) were 18, 147 (15.6%) were 19, 213 (22.5%) were 20, 242 (25.6%) were 21, and 290 (30.7%) were 22 years of age or older.

### 2.2 Measures

All three scales were developed in Turkish. As a result, researchers did not need to do any adaptations to or changes in scales.

#### 2.2.1 Online learning readiness

The online learning readiness scale was developed by Hung et al. (2010) and adapted into Turkish by Yurdugül and Alsancak-Sırakaya (2013). The five-point Likert-type scale has 18 items in 5 sub-dimensions (RMSEA = 0.077; GFI = 0.92; CFI = 0.92; NFI = 0.90). Examples of these items are “I can direct my learning process in the online environment” and “I learn from my mistakes in the online environment.” The Cronbach alpha value of the scale was found to be 0.892.

#### 2.2.2 Student engagement in online learning scale

The scale of university students’ engagement in online learning was originally developed by Sun and Rueda (2012) and adapted into Turkish by Topal et al. (2020). The scale has 19 items in three sub-dimensions (x2/sd: 2.827, GFI: 0.93, AGFI: 0.91, CFI: 0.98, NFI: 0.97, RMSEA: 0.05 and SRMR: 0.06). Examples of the scale items are “I can constantly pay attention to the lesson while taking a course in the online learning environment” in the behavioral sub-dimension; “My studies in the online learning environment excite me” in the affective sub-dimension; and “If I do not know about a concept while taking a course in the online learning environment, I do research on this concept” in the cognitive sub-dimension. The scale responses are five-point Likert-type, and three items are reverse-scored. During data analysis, these items were recoded so that higher scores consistently reflected higher levels of the measured construct. Specifically, responses were reversed as follows: 1 was recoded as 5, 2 as 4, 3 remained the same, 4 as 2, and 5 as 1. Cronbach’s alpha value of the scale was calculated as 0.834.

#### 2.2.3 Online course satisfaction scale

The online course satisfaction scale was developed in Turkish by Bayrak et al. (2020). The scale has 10 items grouped under a single dimension (x2(17) = 61.272, RMSEA = 0.046, GFI = 0.988; CFI = 0.995; NNFI = 0.992). Sample items include items such as “I am satisfied to communicate effectively with my teachers throughout the semester,” and “I am satisfied with the speed of the online system.” Responses to the items are on a five-point Likert scale ranging from 1-strongly disagree to 5-strongly agree. The Cronbach alpha reliability value of the scale was calculated as 0.861.

#### 2.2.4 Control variables

The demographic information collected, namely gender and age, was included in the analysis to examine the effect of exogenous variables on the relationship between online learning readiness, engagement in online learning, and online course satisfaction.

### 2.3 Data analysis

The research data were collected as printed material, and it was observed that there were no missing values among the 945 data collected. Before analyzing the hypotheses, normality and outliers were examined. Skewness and kurtosis values were examined for normality control; skewness and kurtosis values were found to be between −2 and +2, indicating a normal distribution (Kim and Lee, 2020) in all three scales. Accordingly, it is understood that there is no problem with outliers.

After the normality check, the correlation between all variables was first examined in order to test the hypotheses of the research; then the PROCESS macro program (Model 9) was used to control the relationships between the variables in the hypotheses (Hayes, 2022). Path coefficients were interpreted as low (0.10), medium (0.30), and large (0.50) (Cohen, 1988).

## 3 Findings

Table 1 presents mean and standard deviation, namely descriptive statistics, and correlations among variables of the research, which are gender, age, readiness to online learning (ROL), engagement in online learning, and online learning satisfaction. Gender was negatively correlated with readiness to online learning (*r* = − 0.069; *p* < 0.05) while age was positively correlated with readiness to online learning (*r* = 0.067; *p* < 0.05) and engagement in online learning (*r* = 0.099; *p* < 0.01). Readiness to online learning was positively correlated with engagement in online learning (*r* = 0.602; *p* < 0.01) and online learning satisfaction (*r* = 0.450; *p* < 0.01). Besides, engagement in online learning was positively correlated with online learning satisfaction (*r* = 0.497; *p* < 0.01).

**Table 1:** Descriptive statistics and correlations of variables.

Variables	X	Sd	(A)	(B)	(C)	(D)	(E)
(A) Gender	1.34	0.47		0.001	−0.069*	−0.060	−0.044
(B) Age	3.60	1.22	0.001		0.067*	0.099**	0.031
(C) OLR	33.99	7.40	−0.069**	0.067**		0.602**	0.450**
(D) EOL	62.68	11.10	−0.060	0.099**	0.602**		0.497**
(E) OLS	64.85	11.89	−0.044	0.031	0.450**	0.497**	

*N* = 945. Gender: 1 = Female; 2 Male. Age: 1 = 18 years; 2 = 19 years; 3 = 20 years; 4 = 21 years; 5 = 22 + years. OLR, Online Learning Readiness; EOL, Engagement in Online Learning; OLS, Online Learning Satisfaction. *p < 0.05, **p < 0.01.

**Figure 1:**
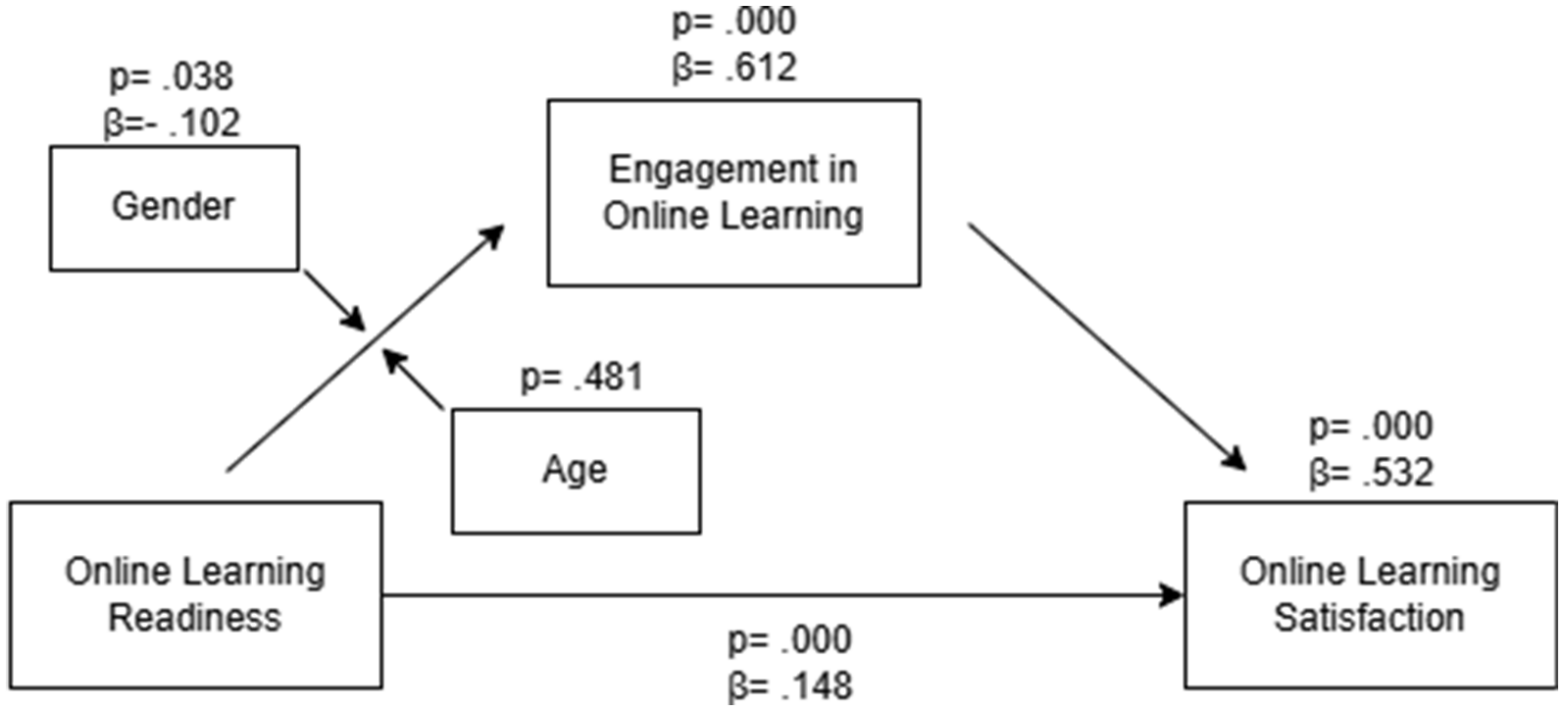
Standardized path coefficients of the hypothesized model.

The hypothesized relationships among online learning readiness, engagement in online learning, and online learning satisfaction were examined using the PROCESS macro in SPSS, and the results were illustrated in Figure 1. The path from online learning readiness to engagement in learning was significant (*β* = 0.612; *p* < 0.001) with a large effect, so Hypothesis 1 was supported. Of the exogenous variables, gender had a significant moderating effect between readiness to online learning and engagement in online learning (*β* = − 0.102; *p* < 0.05) while age did not have it (*p* > 0.05). So, Hypothesis 2 was partly supported. The path from engagement in online learning to online learning satisfaction was significant (*β* = 0.532; *p* < 0.001) and presented a large effect, which supported Hypothesis 3. Thus, the mediating effect of engagement in online learning between online learning readiness and online learning satisfaction stated in Hypothesis 4 was validated.

A boot-strapping approach (5,000 replications) was used to further test the mediating effect of engagement in online learning (Hypothesis 4) as this approach improves the statistical power of mediation analysis (Preacher and Hayes, 2008). As Table 2 shows, the direct effect of online learning readiness on engagement in online learning and engagement in online learning on online learning satisfaction were significant. Besides, the indirect effects of online learning readiness on online learning satisfaction via engagement in online learning was significant. As a result, all hypotheses were supported.

**Table 2:** Direct and indirect effects of variables, and Confidence Intervals (CI).

		Estimated effect (SE)	95% CI
Direct effects			
	OLR → EOL	0.67** (0.12)	[0.44/0.93]
	EOL → OLS	0.23** (0.02)	[0.19/0.28]
	OLR → OLS	0.15** (0.02)	[0.10/0.19]
Indirect effects			
	OLR → Gender → EOL	−0.10** (0.04)	[−0.19/−0.01]
	OLR → Age → EOL	0.01 (0.01)	[−0.02/0.04]
	OLR → EOL → OLS	0.23** (0.03)	[0.19/0.28]

*N* = 945. OLR, Online Learning Readiness; EOL, Engagement in Online Learning; OLS, Online Learning Satisfaction. **p < 0.01.

## 4 Discussion

This study is intended to shed light on the relationship between OLR and OLS, the moderating role of gender and age in the relationship between OLR and EOL, and the mediating role of EOL between OLR and OLS in the EFL context. Following this purpose, data was collected from 945 university students in Türkiye.

As predicted in the first hypothesis, there is a medium level positive significant relationship between OLR and OLS, which could be interpreted as an increase or decrease in one results in the same way as the other. The finding correlates with the existing research suggesting that higher readiness associated with positive satisfaction in online learning environments (Ji et al., 2022; Liu and Kaye, 2016). If one of the reasons of low attendance to online classrooms is low level of OLS and as the finding revealed OLS is significantly correlated with OLR it could be argued that by increasing OLR, the attendance of student to online language classes could be enhanced through skill based trainings towards increasing technical and cognitive capacities of the students related to online learning. In this sense, in today’s digitalized learning environments that lead to increasing number of online courses and increasing enrollment, the orientation trainings intended to enable and sustain students’ OLR might be integrated into the curricula of higher education institutions and need to be revised constantly to respond to the ever-changing requirements caused by rapid developments of technology.

The second hypothesis, which investigates the relationship between OLR and EOL, exposed a high level of positive correlation, which implies that a positive or negative change in OLR results in a similar change in EOL. The literature portrays a wide array of research presenting supporting evidence (Fidan and Koçak-Usluel, 2024; Ji et al., 2022; Kim et al., 2019). In line with the first hypothesis, the efforts and initiatives to support OLR via systematically organized trainings in higher education institutions are assumed to contribute to online learning engagement of students, which could enhance the quality of online EFL learning experience.

Our third hypothesis, which proposes that gender and age moderate the relationship between OLR and EOL, was partially supported since age did not significantly moderate that association. It is possible to find several studies investigating the role of demographic variables as age and gender, suggesting that these variables are associated with OLR and EOL (Laksmiwati et al., 2021; Martin and Bolliger, 2018) or not (Chan et al., 2021; Hung et al., 2010; Tang et al., 2021). Aslam et al. (2021) evidenced no gender difference in relation to OLR and EOL, while age was a source of difference. On the other hand, Laksmiwati et al. (2021) indicated that gender and age were significant sources of difference in terms of OLR and EOL. The present study, distinct from the existing research as it investigated the moderator role of these variables, uncovered that age does not have a moderating role between OLR and EOL, while gender does. We interpreted that age is not significant since the participants come from the same generation, which is labelled as generation Z, who have been extensively exposed to technological tools and developments. As a result, it is not surprising that age is not a significant moderator in terms of the relationshşp between OLR and EOL. It is important to note here is that gender is a significiant demographic variable as revealed in the present research which implies that online learning environments should be respondent to gender specific issues considering the needs, styles and the strategic considerations of the students rather than offering one-size fits-all approach which is widely executed in higher education institutions in Turkiye. Also, in many parts of Turkiye, traditional gender roles may still influence access and use of digital learning tools (Dalgıç-Tetikol et al., 2023). This could be attributed to sociocultural norms that constrain female students’ freedom to engage with technology, contributing to disparities in OLR and OLS.

With respect to the fourth hypothesis, we found a statistically significant medium level of positive relationship between EOL and OLS that is extensively supported by the relevant literature (Baloran et al., 2021; Gray and DiLoreto, 2016; She et al., 2021), which is an expected finding since we argue that engagement in online learning in the EFL context might stimulate satisfaction or vice-versa.

Our last finding, which is considered the first providing empirical evidence that EOL mediates the relationship between OLR and OLS, is significant since it sheds light on the underlying mechanism behind the relationship between these two variables. This could be interpreted as the positive relationship between OLR and OLS could be strengthened by the increase in EOL. In other words, to increase OLS, it is important to support OLR with EOL. This finding provides valuable implications for higher education institutions offering online foreign language courses. Firstly, online learning readiness (OLR) can be effectively enhanced by initiating courses with a diagnostic assessment or readiness checklist designed to evaluate students’ preparedness for online learning. Based on the outcomes of this assessment, educators can provide targeted training sessions or orientation programs focused on the use of digital learning tools and the development of self-directed learning strategies. This approach allows for the identification and support of individual learner needs, thereby fostering greater readiness and improving the likelihood of successful engagement in online learning environments. Second, since EOL has a significant mediating role, higher education institutions should provide multimodal and adaptive online EFL learning environments that support behavioral, cognitive, and emotional engagement of the students through personalized learning content that enables interaction and communication with content, instructor, and peers, which in turn contribute to OLS. Therefore, higher education institutions should provide flexible course structures integrating learning management systems (LMS), providing seamless communication, timely feedback, and real time progress tracking, and incorporating student engagement metrics into course evaluation. Also, online EFL class tutors should provide opportunities for the active engagement of the students because as revealed in the literature learners are more inclined to participate actively when they receive support from teaching staff who are actively involved with students, deeply engaged with the subject matter, and committed to the teaching process (Bryson and Hand, 2007). In this sense, it is crucial for EFL instructors to receive adequate support in achieving these goals in the online language learning environments. We believe that these might be some of the reasons why a very limited number of students attend and engage in the online language classes offered by the university where the present study is conducted. While we advise adaptive, personalized online EFL environments, implementing such systems poses practical, institutional, and pedagogical challenges such as high student-teacher ratios and a lack of professional development, which may prevent instructors from providing the kind of interactive, student-centered experience. Also, this mediation effect may vary depending on learners’ cultural background, motivation for language learning, or access to technological resources—factors that were not fully explored in the current study, which requires further investigation.

This study has certain limitations that need to be acknowledged. First, research data relies on participants’ self-reports, which might threaten the internal validity due to self-report biases. This could be handled in future research by varying the data sources through in depth-interviews, observational records, online system logs, etc. Second, the study sample was based on university students from one university in Türkiye, which limits the generalizability of the findings. Accordingly, it is advised to draw a cross-cultural sample to increase the reliability of the findings. Also, a cross-sectional research design could be considered ineffective for establishing causal relationships. In this respect, future studies might employ longitudinal or experimental designs to offer additional evidence regarding the observed relationships and their underlying mechanisms. Future studies should test our model to investigate OLS as OLS is a comprehensive and multi-dimensional concept and affected by various factors as revealed in the literature.

In conclusion, we assume that EOL is one of the vital components of OLS, together with OLR, and it is significant to investigate engagement deeply for the purpose of presenting a comprehensive understanding of students’ satisfaction in online learning environments.

## Data Availability

The raw data supporting the conclusions of this article will be made available by the authors, without undue reservation. The dataset is available on https://data.mendeley.com/datasets/h8s8vxz3fb/1.
